# Supplementary material for: Human mobility patterns and malaria importation on Bioko Island
Source: Nat Commun. 2019 May 27;10:2332. doi: 10.1038/s41467-019-10339-1 (PMC6536527; doi:10.1038/s41467-019-10339-1)
Supplement: Supplementary file 1 — Supplementary Information [file 41467_2019_10339_MOESM1_ESM.pdf]

HUMAN MOBILITY PATTERNS AND MALARIA IMPORTATION ON BIOKO ISLAND

Guerra et al.

Supplementary Information

Supplementary Note 1: Modeling the Travel Fraction

Since individuals with a history of travel to Río Muni were overall more likely to be infected with malaria, this raises the question of what fraction of people on Bioko were infected as the result of travel to the mainland. Obviously, some of those who had acquired malaria while off-island would remain infected after eight weeks. We know from a variety of sources that the prevalence of malaria declines very slowly in a population, even if there is no further exposure. We utilize a semi-mechanistic model of local exposure, exposure while traveling and travel frequency to explain the patterns that we see in infection prevalence among travelers. Our aim is to explain the travel fraction, defined in the main text as the fraction of the malaria positive population attributable to travel to mainland EG, using our semi-mechanistic model.

We begin by defining our notation:

- PR denotes PfPR - included in our modeled estimates in the main text
- PR_{rm} denotes PfPR among off-island travelers to Río Muni in mainland EG - included in our modeled estimates in the main text
- TP_{rm} denotes the travel prevalence, the proportion of the population who had traveled to Río Muni in mainland EG in the past 8 weeks - included in our modeled estimates in the main text
- PR_{T0} denotes prevalence in the absence of local transmission
- PR_{L0} denotes "local residual transmission", or prevalence in the absence of travel
- TF denotes travel fraction
- h denotes local force of infection (FOI), or the probability of becoming infected, per person, per day
- r denotes the rate that malaria infections clear; we use an exponential model of clearing infections, such that the proportion of a cohort that remains infected t days after becoming infected is e^{-rt} where $r^{-1} = 200$ days
- δ denotes the daily travel return rate, or the number of people who arrive home from travel, per day
- η denotes the proportion of returning travelers who acquired malaria while traveling

At equilibrium, the overall prevalence is

$$\text{PR} = \frac{h + \eta\delta}{h + \eta\delta + r} \tag{1}$$

In the absence of local transmission, $h \to 0$ and the proportion of a population that would be infected as a result of travel would be

$$\text{PR}_{\text{T0}} = \frac{\eta\delta}{\eta\delta + r} \tag{2}$$

The travel fraction is therefore defined as a fraction of total prevalence:

$$\text{TF} = \frac{\text{PR}_{\text{T0}}}{\text{PR}} = \frac{h + \eta\delta + r}{h + \eta\delta}\frac{\eta\delta}{\eta\delta + r} \tag{3}$$

Similarly, local residual transmission quantifies what prevalence would be if travel suddenly ceased:

$$\text{PR}_{\text{L0}} = \frac{h}{h + r} \tag{4}$$

Supplementary Note 2: Daily Travel Rates

We solve for the rate of travel (δ) using the estimated proportion found to have traveled in the study period (8 weeks, or 56 days).

$$\text{TP}_{\text{rm}} = 1 - e^{-\delta \times 56}$$

Solving for δ in terms of the known proportion of travelers:

$$\delta = -\frac{\log(1 - \text{TP}_{\text{rm}})}{56} \tag{5}$$

Supplementary Note 3: Risk of Infection During Travel

In order to estimate η, the probability of returning infected, we consider the difference between the prevalence among travelers (PR_{rm}) and among those who do not travel (P). We use the η to account for the difference $\text{PR}_{\text{rm}} - P$. Additionally, we also assume that travelers recover from infection at an exponential rate, such that the average proportion of returned travelers who are still infected at the time of the survey becomes

$$\eta \times \int_0^{56} e^{-rt} dt$$

Combining these elements allows us to solve for η:

$$\eta = (\text{PR}_{\text{rm}} - P)\frac{56r}{1 - e^{-56r}} \tag{6}$$

There are three values that we use for P, representing the full range of possible values for η. Setting $P = 0$ represents the case where all cases reported among travelers were acquired while off-island,

and defines an upper bound on the range of η. Setting $P = \mathrm{PR}$ represents the case where travelers who leave a Bioko *map-area* are already infected with the same probability as the average person who lives there, and defines a lower bound on the range of η. Lastly, setting $P = \mathrm{PR}_{\mathrm{L0}} = \frac{h}{h+r}$ (eq. 4), the prevalence attributable to local residual transmission, represents the case where the proportion of travelers who leave are already infected due to local residual transmission. This case requires simultaneously solving Supplementary Equations 1 and 6 in order to co-estimate h and η together. We use this last case to formulate our estimates of travel fraction and local residual transmission in the main text (Figure 6).

Note that when η is estimated to be high, h is estimated to be low, and vice-versa. That is to say, the map of malaria prevalence constrains our model such that the cases detected among travelers may either be attributed to local transmission or off-island travel.

Given η it is straightforward to solve for h using Supplementary Equation 1. The final step is to solve for the travel fraction and the local residual transmission using Supplementary Equations 3 and 4.

Supplementary Note 4: Numerical Example

To give an example, consider a *map-area* where the overall prevalence $\mathrm{PR} = .150$, 12% of the population have recently traveled ($\mathrm{TP}_{\mathrm{rm}} = 0.120$), and prevalence among recent travelers is 24% ($\mathrm{PR}_{\mathrm{rm}} = 0.240$).

We use Supplementary Equation 5 to solve for $\delta = 0.00228$. Next we set $P = \frac{h}{h+r}$ and simultaneously solve Supplementary Equations 1 and 6 in order to obtain $h = 4.86 \times 10^{-4}$ and $\eta = .174$. From this, we use Supplementary Equation 3 to find that the travel fraction is $\mathrm{TF} = .489$, and we use Supplementary Equation 4 to find that the local residual transmission gives $\mathrm{PR}_{\mathrm{L0}} = .089$. To obtain the bounds on these estimates, we repeat this procedure by setting $P = 0$ and $P = 0.150$ to estimate that the lower and upper bounds on $\eta = [0.103, 0.275]$ and $h = \left[2.54 \times 10^{-4}, 6.46 \times 10^{-4}\right]$. Consequently, the lower and upper bounds on $\mathrm{PR}_{\mathrm{L0}} = [.048, .115]$ and $\mathrm{TF} = [.300, .744]$.

Supplementary Note 5: Estimates of travel fraction and local residual transmission

Using the methods outlined in the preceding example, we estimate δ, η, h, the travel fraction, and the local residual transmission in each *map-area* on Bioko as well as upper and lower bounds on all quantities. Figure 6 in the main text depicts a map of our estimates of the travel fraction (center)

and local residual transmission (right). Supplementary Figures 1 and 2 show the full range of possible values for travel fraction and local residual transmission.

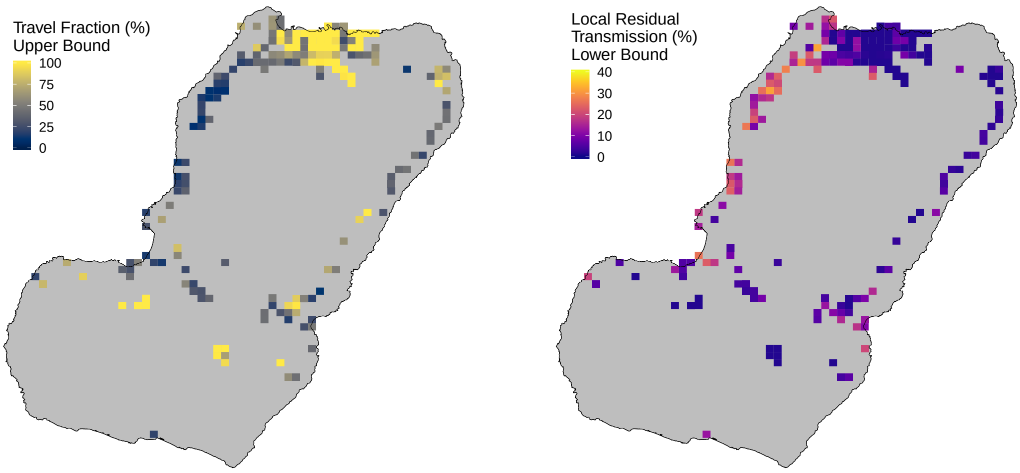

Supplementary Figure 1: Maps of travel fraction lower bound (left) and local residual transmission upper bound (right), estimated using the assumption that the effects of local transmission are maximized.

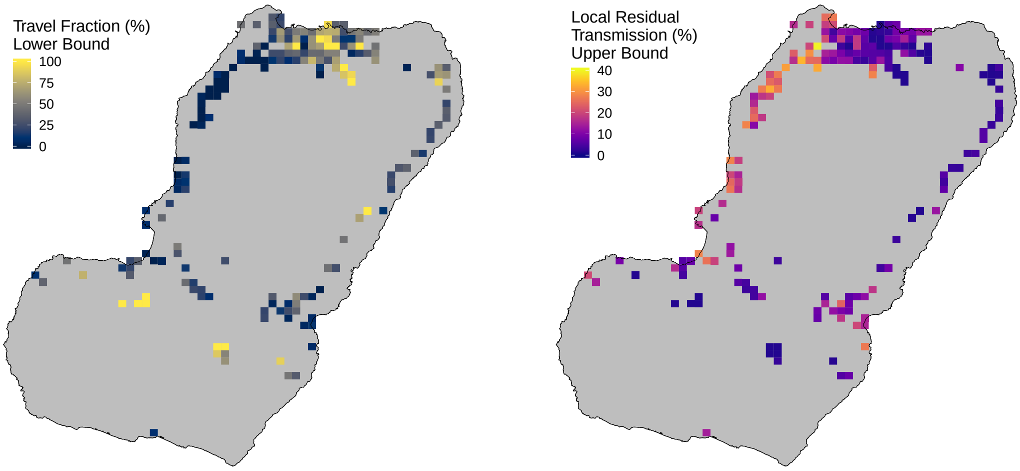

Supplementary Figure 2: Maps of travel fraction upper bound (left) and local residual transmission lower bound (right), estimated using the assumption thta the effects of local transmission are minimized.

We note that our estimates show similar geographical patterns across the full range of our estimates. Travel fraction remains highest in urban Malabo in the north as well as Southern areas, some of which are believed to be at too high an altitude to sustain high transmission. Similarly, local residual transmission is low in urban Malabo and along the Eastern coast and is high along the Northwestern coast.

We may also estimate the fraction of the total population on Bioko who live in areas where importations account for a majority of cases. We use our lower and upper bound estimates of travel fraction to obtain a possible range of travel fraction for each *map-area* and sum over the total number of people living in all *map-areas* with a majority of cases attributable to travel. The results are summarized in Supplementary Table 1.

Supplementary Table 1: Percent of people living in areas where travel fraction is estimated at 100%, and 80% and 50% or more.

Travel fraction	% total population, estimate	% total population, lower bound	% total population, upper bound
**100%**	48.4	9.8	58.3
**80%**	67.2	27.2	70.9
**50%**	74.8	62.7	83.5

Supplementary Note 6: Sensitivity to Travel Data

The travel data analyzed in the main text focused on traveling to mainland EG, and excluded off-island travel to other locations. One possible reason for concern here is that the prevalence and force of infection in Río Muni are very high, such that discounting other off-island travel may bias our estimates of travel fraction and local force of infection.

We test our results' sensitivity to including off-island travel to other destinations. These travel events comprise 15.7% of all off-island travel. Mapping our results in Supplementary Figure 3 and comparing to main text Figure 6 (center and right), we find that the estimated distribution of travel fraction and local residual transmission is largely unchanged. The distribution of people living in areas where travel fraction is 50% or higher is also largely unchanged (Supplementary Table 2).

Supplementary Table 2: Percent of people living in areas where travel fraction is estimated at 100%, and 80% and 50% or more.

Travel fraction	% total population, estimate	% total population, lower bound	% total population, upper bound
**100%**	38.0	10.0	53.6
**80%**	51.9	10.7	69.7
**50%**	72.2	40.9	78.0

Supplementary Note 7: Sensitivity to Treatment

One assumption that our model makes is that on average malaria infections are cleared after $r^{-1} = 200$ days, which assumes that infections are left untreated. It is possible, however, that there is

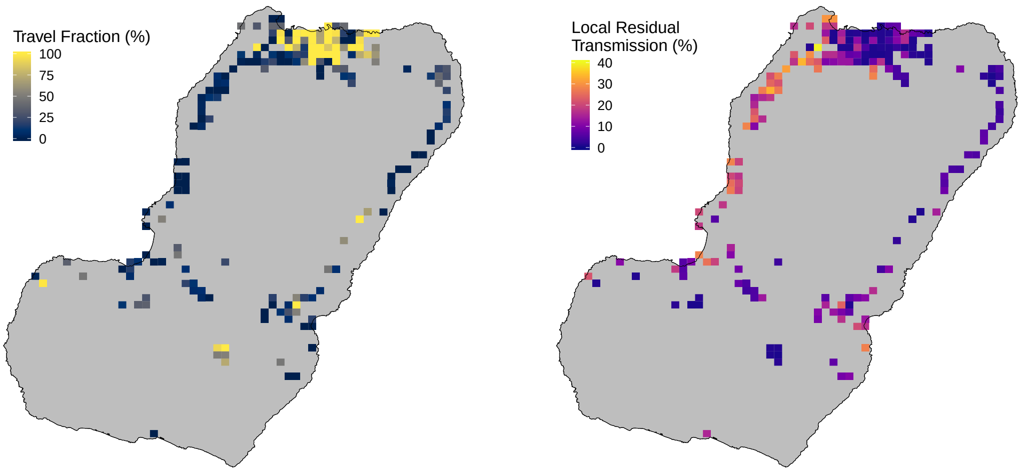

Supplementary Figure 3: Maps of travel fraction (left) and local residual transmission lower bound (right), estimated when including travel to destinations outside of Río Muni. These maps are analogous to the ones shown in the main text Figure 6 (center and right).

sufficient ready access to treatment that individuals are able to clear their infections more quickly. We test the extent to which our model is sensitive to access to treatment, repeating the analysis from the previous section using $r^{-1} = 100$ days. Supplementary Figure 4 depicts two scatter plots that compare how our estimates for travel fraction (left) and local residual transmission (right) change following an increase in the parameter r. Adding treatment that increases the rate at which infections clear decreases our estimates of the travel fraction and slightly increases our estimates of the local residual transmission in some *map-areas*. Future analyses will aim at incorporating data that can provide proxy measures of access to treatment.

Supplementary Note 8: Sensitivity to Travel Frequency Heterogeneity

We perform a basic sensitivity analysis to allowing for heterogeneity in travel frequency. The MIS data include counts of the number of times individuals in each *map-area* have traveled off-island; from this we are able to estimate the mean travel frequency for individuals living in each *map-area*. This averaging implicitly assumes that all individuals travel with equal probability, when it is also possible that some individuals travel more than others.

As an illustrative example, we assume that all travel events estimated from our data are allocated to half as many people who travel twice as frequently. Effectively, for half of the individuals who live in each *map-area* $\text{TP}_{\text{rm}} = 0$ while for the other half $\text{TP}_{\text{rm}} \to 2\text{TP}_{\text{rm}}$. We work in the limit where

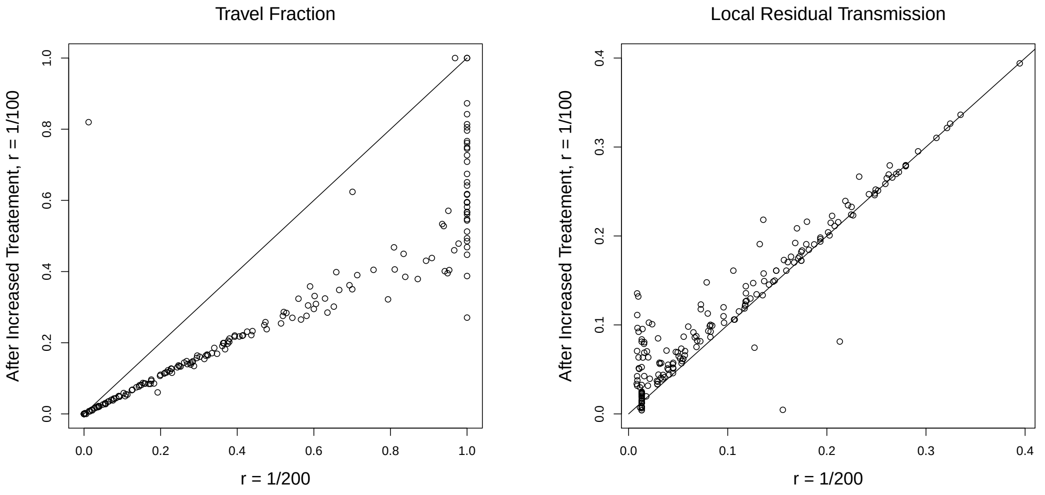

Supplementary Figure 4: Changes to travel fraction and local transmission under different treatment conditions, depicted as scatter plots comparing the two cases ($r = 1/200$ and $r = 1/100$). In both plots, each point represents a different *map-area*.

local transmission is assumed to be minimized ($h \to 0$) such that $\mathrm{PR} = 0$ among non-travelers and $\mathrm{PR} \to \mathrm{PR}_{\mathrm{T0}}$ (Supplementary Equation 2) among travelers.

If we assume that travel is distributed uniformly, $\mathrm{PR} \to \mathrm{PR}_{\mathrm{T0}}$. If we assume that travel is distributed heterogeneously, with half of all individuals traveling twice as frequently, $\mathrm{PR} = \mathrm{PR}_{\mathrm{T0}}/2$. Supplementary Figure 5 shows PR in both cases plotted against one another. The two cases are highly correlated; adding heterogeneity to how travel events are distributed across the population does not appear to dramatically change predicted PfPR.

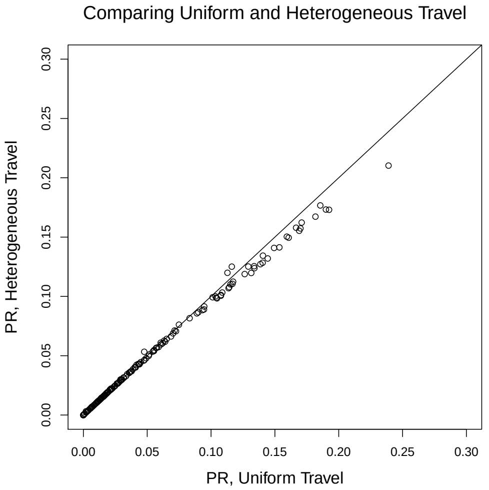

Supplementary Figure 5: Scatter plot of observed PR in the cases of uniformly- and heterogeneously- distributed travel. Each point represents a different *map-area*.

Supplementary Note 9: Credible intervals of covariates and geostatistical model outputs

Supplementary Tables 3 to 8 below summarize the regression coeffcients of predictors selected for each of the models of malaria and travel prevalence. Supplementary Figure 6 shows the uncertainty maps expressed as the 95% credible intervals of the *Pf*PR and TP estimates.

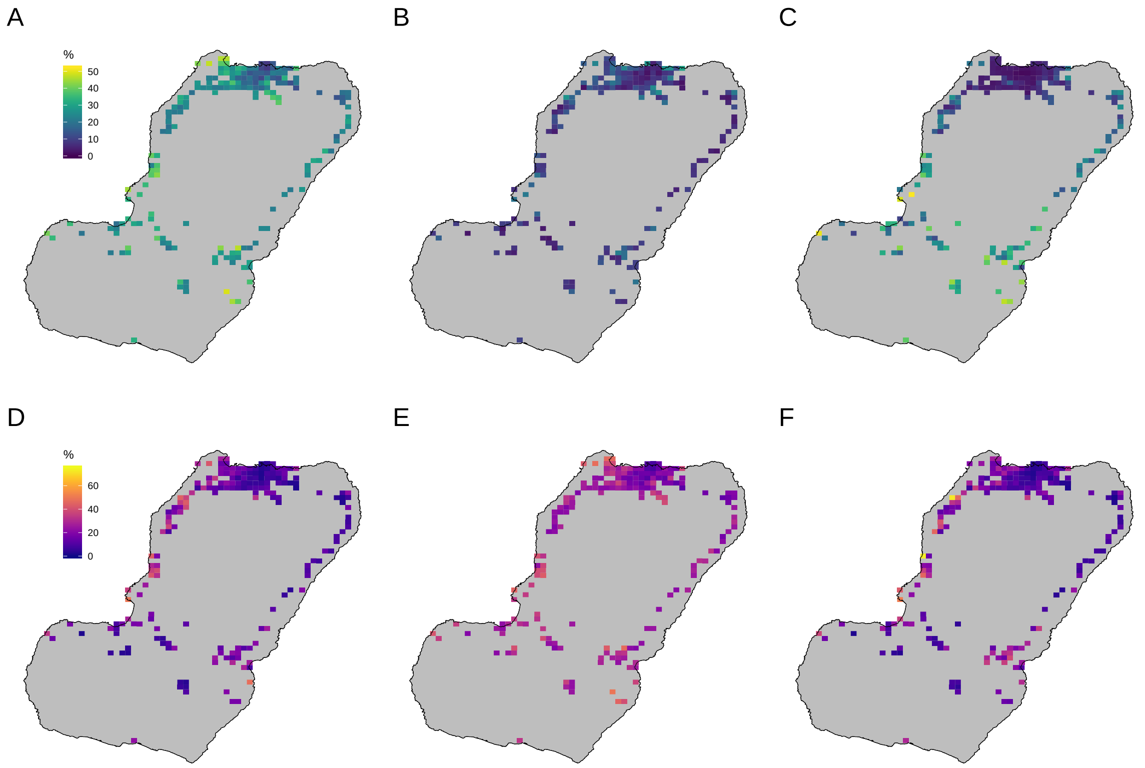

Supplementary Figure 6: Uncertainty maps illustrating the 95% credible intervals for the TP (top row) and *Pf*PR estimates. For TP: A, all individuals, B, travelers to Río Muni, and C, within-island travellers. For *Pf*PR: D, all individuals, E, travelers to Río Muni, and F, non-travelers.

Supplementary Table 3: Regression coefficients of the predictors selected by the final model for prevalence of any travel and their associated 95% Bayesian credible intervals (CI).

Covariate	Mean	Lower 95CI	Upper 95CI
**Intercept**	-1.8525	-2.1349	-1.5707
**Distance to Malabo**	0.0000	0.0000	0.0000
**Population 2015-2016**	0.0000	-0.0003	0.0004

Supplementary Table 4: Regression coefficients of the predictors selected by the final model for prevalence of travel to Río Muni and their associated 95% Bayesian credible intervals (CI).

Covariate	Mean	Lower 95CI	Upper 95CI
**Intercept**	-2.6339	-3.1122	-2.1565
**Distance to Malabo**	0.0001	-0.0003	0.0005
**Population 2015-2016**	0.0000	0.0000	0.0000

Supplementary Table 5: Regression coefficients of the predictors selected by the final model for prevalence of within-island travel and their associated 95% Bayesian credible intervals (CI).

Covariate	Mean	Lower 95CI	Upper 95CI
**Intercept**	-3.5969	-4.0611	-3.1340
**Distance to Malabo**	0.0001	0.0001	0.0001
**Population 2015-2016**	0.0003	0.0003	0.0008

Supplementary Table 6: Regression coefficients of the predictors selected by the final model for *Pf*PR in all individuals and their associated 95% Bayesian credible intervals (CI) .

Covariate	Mean	Lower 95CI	Upper 95CI
**Intercept**	-2.237	-2.725	-1.749
**EVI mean 2015-2016**	3.029	1.578	4.481
**Landcover urban barren**	0.450	0.161	0.737
**Stable Lights 2010**	0.012	0.000	0.025
**TCB mean 2015-2016**	-7.897	-10.762	-5.037
**TCW mean 2015-2016**	-6.252	-11.060	-1.436
**TempSuitabilityPf**	1.917	0.829	3.005

Supplementary Table 7: Regression coefficients of the predictors selected by the final model for *Pf*PR in travelers to Río Muni and their associated 95% Bayesian credible intervals (CI).

Covariate	Mean	Lower 95CI	Upper 95CI
**Intercept**	-0.745	-1.668	0.175
**Accessibility**	0.005	0.002	0.008
**EVI mean 2015-2016**	4.279	1.913	6.644
**Landcover WGS84**	-0.030	-0.061	0.001
**Landcover wetlands**	0.378	-0.011	0.764
**Stable Lights 2010**	0.026	0.014	0.039
**TCB mean 2015-2016**	-7.150	-10.441	-3.868
**TWI**	-0.047	-0.127	0.032

Supplementary Table 8: Regression coefficients of the predictors selected by the final model for *Pf*PR in individuals without history of travel to Río Muni and their associated 95% Bayesian credible intervals (CI).

Covariate	Mean	Lower 95CI	Upper 95CI
**Intercept**	-3.074	-3.673	-2.474
**EVI mean 2015-2016**	1.652	-0.217	3.518
**Landcover urban barren**	0.299	-0.091	0.684
**Landcover water**	1.090	-0.512	2.529
**LST night mean 2015-2016**	0.084	0.025	0.143
**TCB mean 2015-2016**	-5.344	-9.712	-0.976
**Accessibility**	-0.001	-0.004	0.002
**TCW mean 2015-2016**	-3.442	-9.250	2.401
